# The effect of liver metastases on clinical efficacy of first‐line programmed death‐1 inhibitor plus chemotherapy in esophageal squamous cell carcinoma: A post hoc analysis of ASTRUM‐007 and meta‐analysis

**DOI:** 10.1002/cam4.7203

**Published:** 2024-05-21

**Authors:** Jing Gao, Yan Song, Xiaoge Kou, Zhenbo Tan, Shu Zhang, Meili Sun, Jin Zhou, Min Fan, Ming Zhang, Yongxiang Song, Suyi Li, Yuan Yuan, Wu Zhuang, Jingdong Zhang, Li Zhang, Hao Jiang, Kangsheng Gu, Huangyang Ye, Ying Ke, Xiao Qi, Qingyu Wang, Jun Zhu, Jing Huang

**Affiliations:** ^1^ Department of Medical Oncology, National Cancer Center/National Clinical Research Center for Cancer/Cancer Hospital Chinese Academy of Medical Sciences and Peking Union Medical College Beijing China; ^2^ Department of Medical Oncology The First Affiliated Hospital of Xinxiang Medical University Xinxiang China; ^3^ Department of Thoracic Surgery Xingtai People's Hospital Xingtai China; ^4^ Department of Gastrointestinal Oncology Shandong First Medical University Cancer Hospital, Shandong Cancer Hospital Jinan China; ^5^ Department of Medical Oncology Central Hospital Affiliated to Shandong First Medical University Jinan China; ^6^ Department of Medical Oncology Sichuan Cancer Hospital Chengdu China; ^7^ Department of Radiation Oncology Fudan University Shanghai Cancer Center Shanghai China; ^8^ Department of Integrated Traditional and Western Medicine, Shanghai Chest Hospital Shanghai Jiao Tong University Shanghai China; ^9^ Department of Thoracic Surgery Affiliated Hospital of Zunyi Medical University Zunyi China; ^10^ Department of Medical Oncology Anhui Provincial Cancer Hospital Hefei China; ^11^ Department of Medical Oncology Xuzhou Central Hospital Xuzhou China; ^12^ Department of Medical Oncology Fujian Cancer Hospital Fuzhou China; ^13^ Medical Oncology Department of Gastrointestinal Cancer Liaoning Cancer Hospital & Institute, Cancer Hospital of China Medical University Shenyang China; ^14^ Department of Oncology Chongqing University Three Gorges Hospital Chongqing China; ^15^ Department of Radiation Oncology The First Affiliated Hospital of Bengbu Medical College Bengbu China; ^16^ Department of Medical Oncology The First Affiliated Hospital of Anhui Medical University Hefei China; ^17^ Department of Medical Oncology The First Affiliated Hospital of Xiamen University Xiamen China; ^18^ Shanghai Henlius Biotech, Inc. Shanghai China

**Keywords:** anti‐PD‐1 antibody, chemotherapy, esophageal squamous cell carcinoma (ESCC), liver metastases, meta‐analysis, serplulimab

## Abstract

**Objective:**

To explore the efficacy of serplulimab plus chemotherapy in esophageal squamous cell carcinoma (ESCC) patients with liver metastases.

**Methods:**

A post hoc exploratory analysis of ASTRUM‐007 study was performed, focusing on the association between the liver metastases status and the clinical outcomes. A systematic literature search of electronic databases was conducted to identify eligible randomized controlled trials for the meta‐analysis. Study‐level pooled analyses of hazard ratios (HRs) for PFS according to liver metastases were performed.

**Results:**

The post hoc analysis of ASTRUM‐007 showed that although patients with liver metastases had a worse prognosis comparing with the non‐liver metastases patients in both treatment arms (serplulimab plus chemotherapy arm: median PFS, 5.7 vs. 6.6 months, HR 1.57 [95% CI, 1.15–2.13]; median OS, 13.7 vs. 15.3 months, HR 1.48 [95% CI, 1.09–1.98]; placebo plus chemotherapy arm: median PFS, 4.3 vs. 5.5 months, HR 1.58 [95% CI, 1.01–2.39]; median OS, 10.3 vs. 11.2 months, HR 1.32 [95% CI, 0.84–2.00]), OS and PFS benefits derived from serplulimab plus chemotherapy versus placebo plus chemotherapy in this study were observed in both patients with liver metastases (HR of PFS: 0.60; 95% CI, 0.37–0.97; HR of OS: 0.68; 95% CI, 0.43–1.11) and the non‐liver metastases patients (HR of PFS: 0.62; 95% CI, 0.49–0.80; HR of OS: 0.69; 95% CI, 0.55–0.87) with similar magnitude. Three randomized controlled trials were included in the meta‐analysis. Pooled HRs demonstrated that the addition of anti‐PD‐1 antibodies significantly improved PFS compared to chemotherapy alone regardless of liver metastases status.

**Conclusions:**

This study reveals that the presence of liver metastases is a poor prognostic factor but does not affect the improvements in both PFS and OS brought by adding PD‐1 blockade to chemotherapy in ESCC patients. Predictive biomarkers for survival in these patients warrant further investigation.

## 1 INTRODUCTION

Esophageal squamous cell carcinoma (ESCC) is the predominant histological type of esophageal cancer (EC), accounting for approximately 90% of all EC cases worldwide.^1^ The incidence and mortality of ESCC vary across countries and world regions with the highest rates in Eastern Asia and Southern and Eastern Africa.^2^, ^3^ Despite recent advances in treatment strategies for EC, metastatic EC patients generally have poor prognoses, with a median overall survival (OS) of only 8–10 months.^4^, ^5^ One of the most common distant metastatic sites is liver, and the standard treatment for ESCC with liver metastases mainly includes systemic and palliative therapy.^6^ Previous studies have demonstrated that the presence of liver metastases led to inferior survival with the median OS of only 5 months, while that in patients with lung or brain metastases was 6 months.^7^, ^8^, ^9^ Response to chemotherapy remains unsatisfactory. The response rate was only 18% in liver metastases.^10^

In recent years, immune checkpoint inhibitor (ICI) has emerged as a novel treatment option for patients with advanced EC. Anti‐programmed death‐1 (anti‐PD‐1) antibodies combined with chemotherapy have been recommended as the standard‐of‐care for the first‐line treatment in advanced ESCC patients according to the results from multiple randomized phase 3 trials.^11^, ^12^ In a recent multicenter, double‐blind, randomized phase III ASTRUM‐007 trial, we reported that serplulimab (a monoclonal anti‐PD‐1 antibody) plus chemotherapy significantly improved PFS and OS compared with placebo plus chemotherapy in patients with previously untreated, locally advanced, or metastatic ESCC.^13^ However, previous preclinical models revealed that liver metastases could induce immunotherapy resistance through systemic CD8+ T cell deletion.^14^ A few clinical studies have reported that the presence of liver metastases correlated with poor response to immune checkpoint inhibitor in melanoma, urothelial cancer, and non‐small cell lung cancer (NSCLC) patients.^15^, ^16^, ^17^, ^18^, ^19^ As for ESCC, whether patients with baseline liver metastases could still derive clinical benefit from the regimen remains unclear. Evidence on the impact of baseline liver metastases on the efficacy of both chemotherapy and anti‐PD‐1‐chemotherapy combinations in ESCC is limited.^20^

Herein, we performed a post hoc analysis with the data from ASTRUM‐007 to explore the efficacy in subgroups stratified by liver metastases status. In addition, a meta‐analysis according to recently published randomized controlled trials (RCTs) was also conducted to further evaluate the relationship between liver metastases and the efficacy of anti‐PD‐1‐chemotherapy combinations as the first‐line treatment in advanced ESCC patients.

## 2 METHODS

### 2.1 The ASTRUM‐007 study and post hoc analysis

The study design of the randomized, double‐blind, phase III ASTRUM‐007 trial has been reported previously.^13^ Briefly, patients with treatment‐naive, locally advanced or metastatic, PD‐L1‐positive (combined positive score ≥1) ESCC were randomized (2:1) to receive serplulimab (an anti‐PD‐1 antibody) or placebo plus chemotherapy (5‐FU plus cisplatin). In the prespecified final analysis for PFS and interim analysis for OS, the addition of serplulimab to chemotherapy resulted in a significant improvement in both endpoints. For the present study, we included all patients enrolled in the ASTRUM‐007 trial and performed a post hoc analysis according to the absence or presence of liver metastases with updated patient‐level data as of January 9, 2023. Patients included in the study must meet the following liver function criteria: total bilirubin ≤1.5 upper limit of normal (ULN), ALT, AST, and/or ALP≤2.5 ULN; ALT and/or AST≤5 ULN. The efficacy endpoints of the present study were PFS, OS, objective response rate (ORR), and duration of response (DoR) in patients with or without liver metastases. The analyses of all efficacy endpoints except OS were based on the assessments by the blinded independent radiological review committee. The National Cancer Institute Common Terminology Criteria for Adverse Events (v.4.03) were used for classifying and grading adverse events.

### 2.2 Literature search, data extraction, and meta‐analysis

A comprehensive systematic literature search through PubMed, Embase, and Cochrane databases for RCTs (until March 2023) was performed with the following keywords: “randomized controlled trial”, “esophageal squamous cell carcinoma”, and “programmed cell death 1 inhibitor”. Table S1 provides details of the search strategy and inclusion criteria. Two reviewers (G.J. and L.Y.) independently screened and selected the trials for eligibility and extracted information from each trial. The pooled HRs were calculated separately according to the extracted HR of PFS and 95% confidence intervals (CI) from each included study in patients with or without liver metastases to evaluate the efficacy and safety of anti‐PD‐1 agent plus chemotherapy versus conventional chemotherapy. The included RCTs were additionally assessed for risk of bias using the Cochrane Risk of Bias 2 tool, which yielded low risk for all studies included (Figure S1). Stata/MP software version 17.0 (Stata Corporation, College Station, TX, USA) was used to perform all analyses.

### 2.3 Statistical analysis

The median OS, PFS, and DoR for each group were estimated using the Kaplan–Meier approach, with 95% confidence intervals (CIs) calculated using the Brookmeyer‐Crowley method. Between‐group differences in OS, PFS, and DoR were assessed using a log‐rank test. A Cox proportional hazards model was used to estimate the hazard ratios (HRs) between both groups with 95% CI. The ORR and disease control rate (DCR) were presented with 95% CIs (Clopper–Pearson method), and the comparisons between the groups were calculated with the chi‐square test. The follow‐up duration was estimated using the reverse Kaplan–Meier method. All statistical analyses were performed using the SAS software, version 9.4 (SAS Institute Inc., Cary, NC, USA).

## 3 RESULTS

### 3.1 Post hoc subgroup analysis of ASTRUM‐007 according to liver metastases status

#### 3.1.1 Patient characteristics

Of the 551 patients in the ASTRUM‐007 trial, 103 patients had liver metastases at baseline (serplulimab plus chemotherapy, *n* = 71; placebo plus chemotherapy, *n* = 32), and 448 patients were without liver metastases (serplulimab plus chemotherapy, *n* = 297; placebo plus chemotherapy, *n* = 151). Baseline patient characteristics by liver metastases status are presented in Table 1. Of note, the percentage of patients having more than one distant organ metastases was higher in the liver metastases group than in the non‐liver metastases group (81% [*n* = 83] vs. 23% [*n* = 104]). Specifically, bone metastases were more frequently observed in the liver metastases group than in the non‐liver metastases group (25% [*n* = 26] vs. 8% [*n* = 37]). Additionally, 17% (*n* = 75) of patients in the non‐liver metastases group had locally advanced disease, while all patients in the liver metastases group had distantly metastatic disease.

**TABLE 1:** Baseline characteristics by liver metastases status.

Characteristic	Liver metastases		Non‐liver metastases	
	Serplulimab+CF	Placebo+CF	Serplulimab+CF	Placebo+CF
	(*n* = 71)	(*n* = 32)	(*n* = 297)	(*n* = 151)
Median age (range), years	65 (58–68)	64 (58–68)	63 (56–68)	64 (57–68)
Age subgroup				
<65 years	33 (46.5)	16 (50.0)	166 (55.9)	82 (54.3)
≥65 years	38 (53.5)	16 (50.0)	131 (44.1)	69 (45.7)
Sex				
Male	64 (90.1)	27 (84.4)	253 (85.2)	126 (83.4)
Female	7 (9.9)	5 (15.6)	44 (14.8)	25 (16.6)
ECOG perform status				
0	17 (23.9)	9 (28.1)	75 (25.3)	44 (29.1)
1	54 (76.1)	23 (71.9)	222 (74.7)	107 (70.9)
Disease status				
Locally advanced	0 (0.0)	0 (0.0)	46 (15.5)	29 (19.2)
Distantly metastatic	71 (100.0)	32 (100.0)	251 (84.5)	122 (80.8)
Number of metastatic organs				
1	15 (21.1)	5 (15.6)	181 (60.9)	88 (58.3)
≥2	56 (78.9)	27 (84.4)	70 (23.6)	34 (22.5)
Sites of metastases other than liver				
Lymph node	42 (59.2)	23 (71.9)	194 (65.3)	101 (66.9)
Lung	23 (32.4)	9 (28.1)	73 (24.6)	33 (21.9)
Bone	19 (26.8)	7 (21.9)	29 (9.8)	8 (5.3)
PD‐L1 status				
1 ≤ CPS <10	41 (57.7)	18 (56.3)	165 (55.6)	86 (57.0)
CPS ≥10	30 (42.3)	14 (43.7)	132 (44.4)	65 (43.0)
Smoking status				
Current or former smoker	50 (70.4)	21 (65.6)	183 (61.6)	94 (62.2)
Never smoked	21 (29.6)	11 (34.4)	114 (38.4)	57 (37.8)
Drinking status				
Current or former drinker	42 (59.2)	19 (59.4)	179 (60.3)	91 (60.3)
Non‐drinker	29 (40.8)	13 (40.6)	118 (39.7)	60 (39.7)

*Note*: Data are *n* (%) unless otherwise stated. Sex was recorded by the investigators according to the identity information provided by the patients.

Abbreviations: CF, cisplatin and 5‐fluorouracil; CPS, combined positive score; ECOG, Eastern Cooperative Oncology Group; PD‐L1, programmed death ligand 1.

#### 3.1.2 Association of liver metastases with survival

**FIGURE 1:**
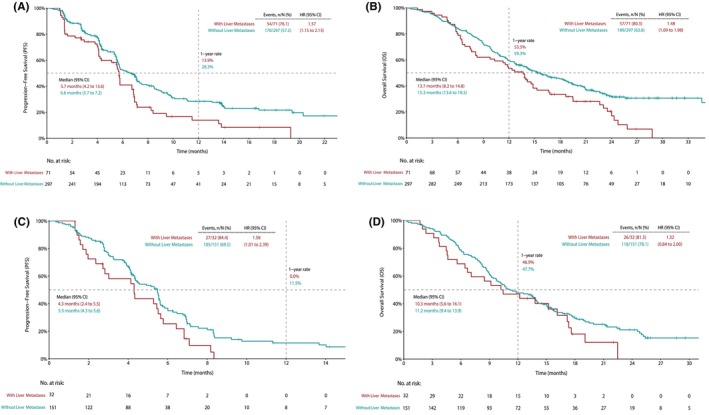
The post hoc analysis of the ASTRUM‐007 study according to treatment regimens. Kaplan–Meier estimates of (A) progression‐free survival (PFS) and (B) overall survival (OS) in patients receiving serplulimab‐chemotherapy treatment; Kaplan–Meier estimates of (C) PFS and (D) OS in patients receiving placebo‐chemotherapy treatment. Patients with liver metastases are shown in red, whereas those without liver metastases are shown in green. CI, confidence interval; HR, hazard ratio.

At the data cutoff date of January 9, 2023, the median follow‐up duration was 24.2 months for patients in the serplulimab plus chemotherapy group and 24.3 months in the placebo plus chemotherapy group. In the patients randomized to the serplulimab plus chemotherapy arm, liver metastases were associated with inferior OS and PFS (Figure. 1). The median PFS was 5.7 months (95% CI, 4.2–13.6) in patients with liver metastases and 6.6 months (95% CI, 5.7–7.2) in those without liver metastases (HR: 1.57 [95% CI, 1.15–2.13], *p* = 0.0038; Figure. 1A). The median OS was 13.7 months (95% CI, 8.2–14.8) in patients with liver metastases and 15.3 months (95% CI, 13.6–18.5) in those without liver metastases (HR: 1.48 [95% CI, 1.09–1.98], *p* = 0.0096; Figure. 1B). Likewise, in patients who received placebo plus chemotherapy, liver metastases were also associated with shorter PFS (median PFS 4.3 months vs. 5.5 months, HR: 1.58 [95% CI, 1.01–2.39], *p* = 0.0357; Figure. 1C) and OS (median OS 10.3 months vs. 11.2 months, HR: 1.32 [95% CI, 0.84–2.00], *p* = 0.2009; Figure. 1D). No statistically significant association between liver metastases and ORR was observed in patients of either treatment arm. The ORR in patients with or without liver metastases was 57.8% versus 57.9% (odds ratio [OR]: 0.99 [95% CI, 0.59–1.68], *p* = 0.9797) in the serplulimab plus chemotherapy arm, and 46.9% versus 41.1% (OR: 1.27 [95% CI, 0.59–2.73], *p* = 0.5461) in the placebo plus chemotherapy arm (Table 2). DoR in patients with or without liver metastases was 5.4 months versus 7.7 months in the serplulimab plus chemotherapy arm, and 4.2 months versus 5.1 months in the placebo plus chemotherapy arm (Table 2).

**TABLE 2:** Antitumor response assessed by independent radiological review committee in patients with or without baseline liver metastases.

	Liver metastases		Non‐liver metastases	
	Serplulimab+CF (*n* = 71)	Placebo+CF (*n* = 32)	Serplulimab+CF (*n* = 297)	Placebo+CF (*n* = 151)
Best overall response^a^				
Complete response	3 (4.2)	1 (3.1)	46 (15.5)	11 (7.3)
Partial response	38 (53.5)	14 (43.8)	126 (42.4)	51 (33.8)
Stable disease	13 (18.3)	4 (12.5)	67 (22.6)	58 (38.4)
Progressive disease	15 (21.1)	8 (25.0)	34 (11.4)	20 (13.2)
Not evaluable or not available	2 (2.8)	5 (15.6)	24 (8.1)	11 (7.3)
Objective response rate^b^				
% (95% CI)	57.7 (45.5–69.4)	46.9 (29.1–65.3)	57.9 (52.1–63.6)	41.1 (33.1–49.4)
*p* value	0.3077		0.0007	
Disease control rate^c^				
% (95% CI)	76.1 (64.5–85.4)	59.4 (40.6–76.3)	80.5 (75.5–84.8)	79.5 (72.1–85.6)
*p* value	0.0862		0.8020	
Duration of response^d^, months				
Median (95% CI)	5.4 (4.2–6.9)	4.2 (3.0–5.3)	7.7 (5.9–9.2)	5.1 (4.2–5.7)

*Note*: Data are *n* (%) unless otherwise indicated. Antitumor response was assessed per the Response Evaluation Criteria in Solid Tumors version 1.1.

Abbreviations: CF, cisplatin and 5‐fluorouracil; CI, confidence interval.

^a^ Percentages may not add up to 100% due to rounding.

^b^ Objective response rate was defined as proportion of patients achieving complete or partial response.

^c^ Disease control rate was defined as proportion of patients achieving complete or partial response, or stable disease.

^d^ Duration of response was assessed in patients who achieved complete or partial response and defined as time from first objective response to disease progression or death from any cause.

#### 3.1.3 Association between liver metastases and the benefit of adding serplulimab

**FIGURE 2:**
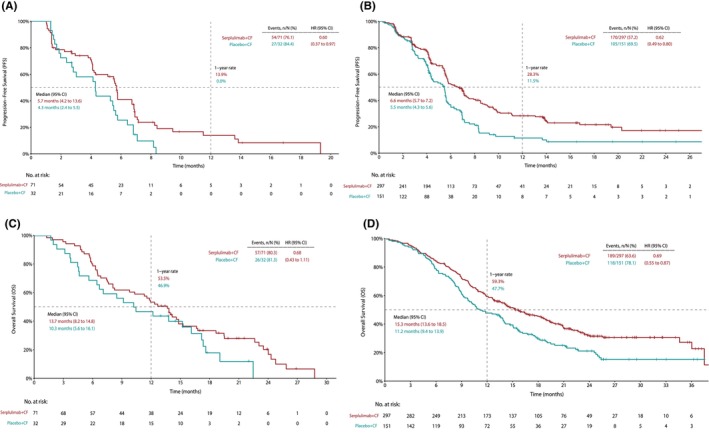
The post hoc analysis of the ASTRUM‐007 study according to liver metastases status. Kaplan–Meier estimates of progression‐free survival (PFS) in patients (A) with and (B) without liver metastases; overall survival (OS) in patients (C) with and (D) without liver metastases. Patients receiving serplulimab‐chemotherapy treatment are shown in red, whereas those receiving placebo‐chemotherapy treatment are shown in green. CF, cisplatin and 5‐fluorouracil; CI, confidence interval; HR, hazard ratio.

Serplulimab plus chemotherapy significantly prolonged PFS compared with placebo plus chemotherapy in both the liver metastases and non‐liver metastases subgroups. In patients with liver metastases, the median PFS was 5.7 months (95% CI, 4.2–13.6) in the serplulimab plus chemotherapy arm versus 4.3 months (95% CI, 2.4–5.5) in the placebo plus chemotherapy arm, with an unstratified HR of 0.60 (95% CI, 0.37–0.97; *p* = 0.0300) (Figure 2A). Similarly, in patients without liver metastases, the median PFS was 6.6 months (95% CI, 5.7–7.2) in the serplulimab plus chemotherapy arm versus 5.5 months (95% CI, 4.3–5.6) in the placebo plus chemotherapy arm with an unstratified HR of 0.62 (95% CI, 0.49–0.80; *p* = 0.0001) (Figure 2B). The 1‐year PFS rates of the serplulimab plus chemotherapy arm versus the placebo plus chemotherapy arm were 13.9% versus 0% in patients with liver metastases, and 28.3% versus 11.5% in patients without liver metastases.

As for OS, the addition of serplulimab to chemotherapy significantly improved OS in patients without liver metastases. The median OS was 15.3 months (95% CI, 13.6–18.5) in the serplulimab plus chemotherapy arm versus 11.2 months (95% CI, 9.4–13.9) in the placebo plus chemotherapy arm, with an unstratified HR of 0.69 (95% CI, 0.55–0.87; *p* = 0.0012) (Figure 2D). However, in patients with liver metastases (Figure 2C), OS tended to be longer in the serplulimab plus chemotherapy arm, but did not have statistical significance. The median OS was 13.7 months (95% CI, 8.2–14.8) in the serplulimab plus chemotherapy arm versus 10.3 months (95% CI, 5.6–16.1) in the placebo plus chemotherapy arm, with an unstratified HR of 0.68 (95% CI, 0.43–1.11; *p* = 0.1103). The 1‐year OS rates of the serplulimab plus chemotherapy arm versus the placebo plus chemotherapy arm were 53.5% versus 46.9% in patients with liver metastases, and 59.3% versus 47.7% in patients without liver metastases. The similar HRs with PFS and OS observed in the two subgroups indicated that the magnitude of survival benefits from the addition of serplulimab to chemotherapy was not affected by liver metastases status.

#### 3.1.4 PFS and OS in different liver metastases status stratified by PD‐L1 expression

In the CPS≥10 subgroup, patients with liver metastases receiving serplulimab combined with chemotherapy demonstrated a 54% reduction in the risk of disease progression (median PFS: 6.9 vs. 5.3 months, HR 0.46 [95% CI, 0.21–1.02], *p* = 0.0418) and a 58% reduction in the risk of death (median OS: 13.8 vs. 6.8 months, HR 0.42 [95% CI, 0.21–0.90], *p* = 0.0167) compared to placebo plus chemotherapy (Figures S2A and S3A). These improvements surpassed those observed in patients without liver metastases (median PFS: 7.3 vs. 5.5 months, HR 0.54 [95% CI, 0.37–0.78], *p* = 0.0008; median OS: 19.1 vs. 13.9 months, HR 0.61 [95% CI, 0.43–0.88], *p* = 0.0071) (Figures S2B and S3B). In 1≤CPS<10 subgroup, serplulimab combined with chemotherapy did not reduce the risk of disease progression (median PFS: 4.3 vs. 4.3 months, HR 0.76 [95% CI, 0.42–1.42], *p* = 0.3759) or mortality (median OS: 11.9 vs. 15.2 months, HR 0.94 [95% CI, 0.50–1.84], *p* = 0.8495) in patients with liver metastases (Figures S2C and S3C).

#### 3.1.5 Liver metastases and safety

We further compared the safety profiles of the two treatment arms in patients with liver metastases (*n* = 103) (Table 3). Among them, 75 were allocated to the serplulimab plus chemotherapy arm, and 28 were in the placebo plus chemotherapy arm. Treatment‐related adverse events of any grade were observed in 100.0% (75/75) of patients in the serplulimab plus chemotherapy arm versus 96.4% (27/28) of patients in the placebo plus chemotherapy arm, whereas grade 3–4 events were reported in 58.7% (44/75) of patients and 50.0% of patients (14/28), respectively. The incidence of hepatic AEs was higher in the serplulimab plus chemotherapy arm than that in the placebo plus chemotherapy arm. Specifically, the incidence of aspartate aminotransferase (AST) increased, alanine aminotransferase (ALT) increased, and blood bilirubin increased were 24.0% (18/75) versus 3.6% (1/28), 14.7% (11/75) versus 7.1% (2/28), and 10.7% (8/75) versus 7.1% (2/28) in the serplulimab plus chemotherapy arm versus placebo plus chemotherapy arm, respectively.

**TABLE 3:** Summary of adverse events in patients with liver metastases.

Events, *n* (%)	Serplulimab+CF (*n* = 75^a^)		Placebo+CF (*n* = 28)	
	Any grade	Grade 3–4	Any grade	Grade 3–4
Any treatment‐related AEs	75 (100.0)	44 (58.7)	27 (96.4)	14 (50.0)
Treatment‐related AEs leading to death	5 (6.7)		2 (7.1)	
Treatment‐related AEs in ≥5% of patients				
Anemia	59 (78.7)	15 (20.0)	22 (78.6)	5 (17.9)
Nausea	43 (57.3)	2 (2.7)	18 (64.3)	0
White blood cell count decreased	44 (58.7)	6 (8.0)	15 (53.6)	3 (10.7)
Neutrophil count decreased	38 (50.7)	11 (14.7)	14 (50.0)	4 (14.3)
Vomiting	28 (37.3)	3 (4.0)	9 (32.1)	1 (3.6)
Appetite decreased	22 (29.3)	1 (1.3)	12 (42.9)	0
Platelet count decreased	25 (33.3)	2 (2.7)	7 (25.0)	0
Asthenia	18 (24.0)	1 (1.3)	11 (39.3)	0
Aspartate aminotransferase increased	18 (24.0)	2 (2.7)	1 (3.6)	0
Weight decreased	12 (16.0)	0	3 (10.7)	0
Alanine aminotransferase increased	11 (14.7)	3 (4.0)	2 (7.1)	1 (3.6)
Blood creatinine increased	11 (14.7)	3 (4.0)	2 (7.1)	1 (3.6)
Hyponatremia	7 (9.3)	2 (2.7)	6 (21.4)	1 (3.6)
Hypoalbuminemia	9 (12.0)	0	2 (7.1)	0
Lymphocyte count decreased	9 (12.0)	3 (4.0)	1 (3.6)	0
Proteinuria	9 (12.0)	0	1 (3.6)	0
Blood bilirubin increased	8 (10.7)	3 (4.0)	2 (7.1)	1 (3.6)
Constipation	6 (8.0)	0	4 (14.3)	0
Hypothyroidism	5 (6.7)	0	3 (10.7)	0
Hypercholesterolemia	7 (9.3)	0	0	0
γ‐glutamyltransferase increased	5 (6.7)	2 (2.7)	2 (7.1)	0
Hyperthyroidism	5 (6.7)	0	1 (3.6)	0

*Note*: The safety set included all patients with liver metastasis who received at least one dose of study treatment. Adverse events were recorded from the first dose of study treatment until 90 days after the last dose or the start of new antitumor treatment, whichever occurred first.

Abbreviations: AE, adverse event; CF, cisplatin and 5‐fluorouracil.

^a^ Inclusive of four patients who were randomized to receive placebo plus chemotherapy, but received serplulimab plus chemotherapy due to an error in drug distribution.

### 3.2 Meta‐analysis

#### 3.2.1 Study selection and the eligible trials

**FIGURE 3:**
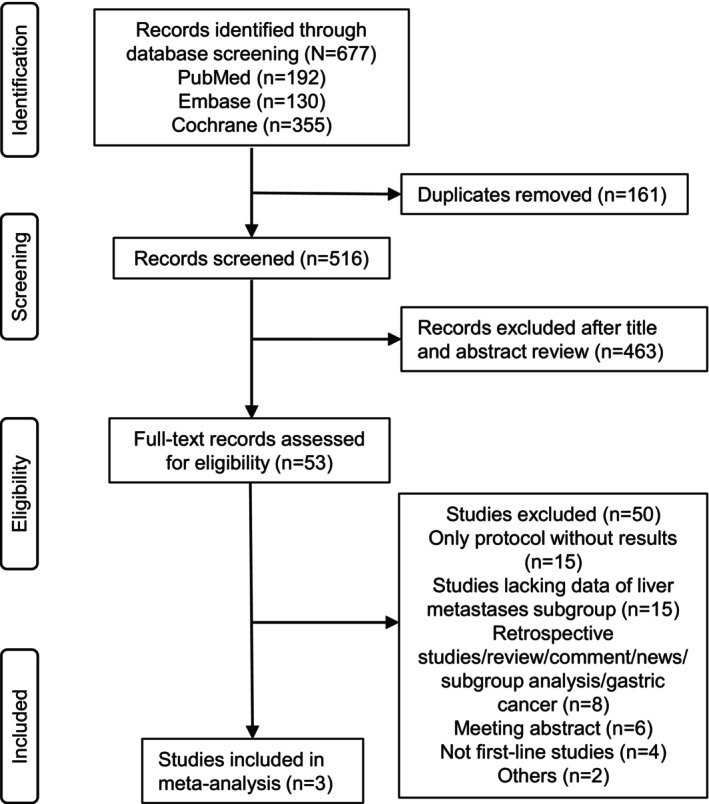
Flow diagram of study inclusions and exclusions.

A total of 677 records were retrieved from the databases through the search strategies shown in the Appendix S1. The procedures of how studies were identified and screened are shown in a flowchart (Figure 3). Three eligible phase III RCTs investigating the efficacy of anti‐PD‐1 antibodies plus chemotherapy stratified by the presence or absence of liver metastases were included: ESCORT‐1st, ASTRUM‐007, and ORIENT‐15, and a total of 1806 patients were included in the meta‐analysis. No significant heterogeneity between studies was detected in the heterogeneity assessment (PFS: *I* ^2^ = 0.0%, *p* = 0.963), and therefore the fixed‐effects model was deployed to calculate the pooled effects on PFS in the data analysis.

#### 3.2.2 Efficacy by liver metastases status

Our meta‐analysis showed that anti‐PD‐1 antibodies plus chemotherapy significantly improved PFS compared to chemotherapy alone in the first‐line treatment of patients with liver metastases of ESCC. The pooled HR for PFS was 0.60 (95% CI, 0.48–0.76). Consistent clinical benefits with the addition of PD‐1 inhibitors to chemotherapy were also observed in patients without liver metastases, as the pooled HR was 0.57 (95% CI, 0.50–0.65) for PFS in these patients (Figure S4).

Concerning the magnitude of clinical benefits from the addition of PD‐1 inhibitors to chemotherapy in patients with or without liver metastases, no statistical differences in PFS advantage (HR 0.60 vs. 0.57, *p* = 0.659) were observed between the two subgroups.

## 4 DISCUSSION

Based on the phase III ASTRUM‐007 study, serplulimab plus chemotherapy is superior to placebo plus chemotherapy and has become a new option for the first‐line treatment of patients with PD‐L1 positive, locally advanced, or metastatic ESCC. In the present study, our post hoc subgroup analysis of ASTRUM‐007 revealed that patients with baseline liver metastases derived PFS and OS benefits from the addition of serplulimab to chemotherapy, although these patients had shorter PFS and OS compared with those without liver metastases, regardless of their treatment allocation.

There were two studies which suggested that the presence of liver metastases was an unfavorable prognostic factor in patients with esophageal cancer including both squamous cell carcinoma and adenocarcinoma.^21^, ^22^ A large cohort‐based study demonstrated that esophageal cancer patients with liver metastases had shortened OS compared with those without liver metastases (6.00 months vs. 15.00 months).^21^ In another cohort of patients with stage IV esophageal cancer from the Surveillance, Epidemiology, and End Results (SEER) database, liver metastasis was identified as one of the independent risk factors for early death (defined as death within 3 months of diagnosis).^22^ Of note, these previous studies were performed before immune checkpoint inhibitors were used in advanced esophageal cancer patients. In our present study, ESCC patients with baseline liver metastases had inferior PFS and OS compared with those without liver metastases in both treatment arms. Specifically, patients with and without baseline liver metastases had similar ORRs, while in the subgroup with baseline liver metastases, we observed a higher proportion of patients with a best response of PD (21.1% vs. 11.4% in serplulimab plus chemotherapy arm), indicative of an increased likelihood of primary resistance to treatment. Meanwhile, the duration of response among patients with liver metastases was also shorter. Our findings suggested that in the era of immuno‐oncology, liver metastases remained a negative prognostic factor for advanced ESCC.

Previous studies have shown that liver metastasis was associated with reduced efficacy of immune checkpoint inhibition alone in several cancer types, including melanoma, NSCLC, renal cell carcinoma, and microsatellite instability‐high (MSI‐H) or mismatch repair deficient (dMMR) colorectal cancer.^23^, ^24^, ^25^, ^26^ The inferior response or survival outcomes might result from immune suppression induced by liver metastases, generating CD11b + macrophages to lower CD8+ T cells, and causing systemic regulatory T‐cell activation.^14^, ^27^ Given that the ability to enhance the immune response by anti‐PD‐1 monotherapy might be partly counteracted in patients with liver metastases, whether the addition of PD‐1 blockade to chemotherapy, as investigated in the ASTRUM‐007 study, could provide meaningful benefits in this subpopulation became an important question to aid clinical decision‐making.

The findings of our post hoc analyses suggested that despite an unfavorable prognosis, advanced ESCC patients with liver metastases had improved PFS with the addition of serplulimab to chemotherapy, whereas the tendency of OS prolongation did not reach statistical significance (median OS 13.7 months vs. 10.3 months, HR 0.68, 95% CI, 0.43–1.11), partly due to the limited number of patients with baseline liver metastases in this single study. In our meta‐analysis with a considerably larger sample size, we observed significant improvements in PFS with PD‐1 inhibition plus chemotherapy compared with chemotherapy alone in patients with baseline liver metastases. Meanwhile, we also showed that PD‐1 inhibitors plus chemotherapy provided similar magnitude of survival benefit for patients with or without liver metastases, as characterized by the similar HRs with PFS (HR 0.60 vs. 0.57, *p* = 0.659). Thus, the poorer outcomes of patients with liver metastases treated with immune checkpoint inhibitor monotherapy seemed to have been reversed with the incorporation of chemotherapy. The underlying mechanistic explanation for the observation might be that the induction of immunogenic cell death by platinum‐based chemotherapy could recruit dendritic cells, downregulate PD‐L1 and PD‐L2, and enhance tumor‐specific T‐cell activation.^28^, ^29^ Taken together, these findings further supported the application of this combination strategy in this patient subgroup.

According to subgroup analysis, patients with CPS≥10 and liver metastases can significantly benefit from chemotherapy combined with immunotherapy in PFS and OS, suggesting that chemotherapy combined with immunotherapy is the optimal first‐line treatment choice for these patients. However, in patients with 1≤CPS<10 and liver metastases, the improvement of PFS and OS was not observed. These subgroup analyses have shown that the ESCC patients with liver metastases and with higher PD‐L1 expression may derive more favorable PFS and OS from the addition of serplulimab than their counterparts with low PD‐L1 expression.

Although rates of treatment‐related hepatic adverse events were slightly higher in serplulimab plus chemotherapy treated patients with baseline liver metastases than in the overall ASTRUM‐007 study population, they were primarily of grade 1–2, and other treatment‐related adverse events were comparable between the two treatment groups among patients with liver metastases. We may therefore conclude that adverse events with PD‐1 inhibition plus chemotherapy were manageable in this subpopulation, without new safety signals. The patients included in the study were all thoroughly evaluated and had sufficient liver functional, the proportion of patients with liver metastases was essentially consistent between the experimental group and the control group, which ensuring the safety of the research and the reliability of its conclusions. In real‐world applications, patients' conditions will be more complex, and the choice of treatment will need to be individually tailored to each patient's circumstances.

Our analysis had some limitations. Due to the post hoc, exploratory nature of the subgroup analyses, our results were not powered with adequate statistical rigor, and therefore should be interpreted with caution. The findings require further investigation, and mechanistic and additional confirmatory studies are warranted.

## 5 CONCLUSIONS

In summary, although liver metastases were associated with inferior OS and PFS in both patients treated with PD‐1 inhibitors plus chemotherapy and chemotherapy alone, the combination of anti‐PD‐1 antibodies with chemotherapy is an effective treatment option for previously untreated advanced ESCC patients with liver metastases. Efforts are warranted to better identify clinical characteristics and biomarkers of survival and to further investigate the tumor microenvironment within the liver to improve treatment efficacy in this subpopulation.

## AUTHOR CONTRIBUTIONS

**Jing Gao:** Conceptualization (equal); data curation (equal); formal analysis (equal); methodology (equal); resources (equal); software (equal); writing – original draft (lead); writing – review and editing (equal). **Yan Song:** Investigation (equal); resources (equal); writing – review and editing (equal). **Xiaoge Kou:** Investigation (equal); resources (equal); writing – review and editing (equal). **Zhenbo Tan:** Investigation (equal); resources (equal); writing – review and editing (equal). **Shu Zhang:** Investigation (equal); resources (equal); writing – review and editing (equal). **Meili Sun:** Investigation (equal); resources (equal); writing – review and editing (equal). **Jin Zhou:** Investigation (equal); resources (equal); writing – review and editing (equal). **Min Fan:** Investigation (equal); resources (equal); writing – review and editing (equal). **Ming Zhang:** Investigation (equal); resources (equal); writing – review and editing (equal). **Yongxiang Song:** Investigation (equal); resources (equal); writing – review and editing (equal). **Suyi Li:** Investigation (equal); resources (equal); writing – review and editing (equal). **Yuan Yuan:** Investigation (equal); resources (equal); writing – review and editing (equal). **Wu Zhuang:** Investigation (equal); resources (equal); writing – review and editing (equal). **Jingdong Zhang:** Investigation (equal); resources (equal); writing – review and editing (equal). **Li Zhang:** Investigation (equal); resources (equal); writing – review and editing (equal). **Hao Jiang:** Investigation (equal); resources (equal); writing – review and editing (equal). **Kangsheng Gu:** Investigation (equal); resources (equal); writing – review and editing (equal). **Huangyang Ye:** Investigation (equal); resources (equal); writing – review and editing (equal). **Ying Ke:** Project administration (equal); software (equal); supervision (equal); writing – review and editing (equal). **Xiao Qi:** Methodology (lead); software (lead); writing – review and editing (equal). **Qingyu Wang:** Project administration (equal); supervision (equal); writing – review and editing (equal). **Jun Zhu:** Project administration (equal); supervision (equal); writing – review and editing (equal). **Jing Huang:** Conceptualization (equal); funding acquisition (equal); investigation (equal); project administration (equal); resources (lead); supervision (lead); writing – original draft (equal); writing – review and editing (lead).

## FUNDING INFORMATION

The study was partly supported by the Major Project of Medical Oncology Key Foundation of Cancer Hospital Chinese Academy of Medical Sciences (Grant No. CICAMS‐MOMP2022001 to J.H.).This study was supported by Shanghai Henlius Biotech, Inc.

## CONFLICT OF INTEREST STATEMENT

Ying Ke, Xiao Qi, Qingyu Wang, and Jun Zhu are employees of Shanghai Henlius Biotech, Inc.

## ETHICS STATEMENT

The study protocol and all amendments were approved by the institutional review board/ethics committee at each site. The ethics committee of the leading clinical center was the Ethics Committee of National Cancer Center/Cancer Hospital, Chinese Academy of Medical Sciences and Peking Union Medical College.

## PATIENT CONSENT STATEMENT

All patients provided written informed consent prior to study participation.

## CLINICAL TRIAL REGISTRATION

The present study is a post hoc analysis of the ASTRUM007 trial. This trial was registered with ClinicalTrials.gov (NCT03958890).

## Supporting information


Appendix S1.

## Data Availability

The data that support the findings of this study are available from the corresponding author upon reasonable request. All relevant data are provided within this manuscript and supporting files, or within the files for the previous study publications by Yan Song, et al. *Nat Med*. 2023 Feb;29(2):473–482 and Jing Huang, et al. *J Clin Oncol* 41, 2023 (suppl 16; abstr e16016).
