# Supplementary material for: The effect of liver metastases on clinical efficacy of first‐line programmed death‐1 inhibitor plus chemotherapy in esophageal squamous cell carcinoma: A post hoc analysis of ASTRUM‐007 and meta‐analysis
Source: Cancer Med. 2024 May 21;13(10):e7203. doi: 10.1002/cam4.7203 (PMC11106639; doi:10.1002/cam4.7203)
Supplement: Supplementary file 1 — Appendix S1. [file CAM4-13-e7203-s001.docx]

**Supplementary material**

| **Supplemental Method** | |
| --- | --- |
| **Supplemental Table 1.** Search strategy | |
| **Supplementary Figure 1** | Risk-of-bias assessment of the three included RCTs. |
| **Supplementary Figure 2** | PFS in the PD-L1 expression subgroups of patients with or without liver metastases. |
| **Supplementary Figure 3** | OS in the PD-L1 expression subgroups of patients with or without liver metastases. |
| **Supplementary Figure 4** | Meta-analysis of PFS with PD-1 antibody plus chemotherapy versus chemotherapy alone in the liver metastases and non-liver metastases subgroups. |

**Supplementary Method**

The full search strategy is detailed as below.

Inclusion criteria included reports of phase III randomized controlled trials (RCTs) investigating the benefit of adding anti-PD-1 antibody to chemotherapy versus chemotherapy alone (or with placebo-controlled) in the first-line treatment of patients with advanced esophageal squamous cell carcinoma (ESCC).

Exclusion criteria included phase I, phase II, dose-finding, adjuvant and neoadjuvant, second or later-line setting trials; news, editorials, letters, commentaries, retrospective studies, review articles; trials not reporting the hazard ratios (HRs) of overall survival or progression-free survival according to the presence or absence of liver metastases.

Preferred Reporting Items for Systematic Reviews and Meta-analysis (PRISMA) reporting guideline was used to conduct this meta-analysis including relevant randomized clinical trials (RCTs). Two authors (G.J. and L.Y.) independently screened the trials for eligibility and extracted the following information from each trial: trial name, year of publication, sample size, HR in both patients with liver metastases and patients without liver metastases. The included RCTs were additionally assessed for risk of bias using the Cochrane risk-of-bias (RoB 2) tool, which yielded low risk for all studies included (Supplementary Figure 1 in Additional file 2)

**Supplementary Table 1. Search strategy**

|  | Search strategy | Number of studies |
| --- | --- | --- |
| PubMed | ("randomized"[Title/Abstract] OR "randomly"[Title/Abstract] OR "trial"[Title/Abstract] OR "placebo"[Title/Abstract] OR "rct"[Title/Abstract] OR "controlled clinical trial"[Publication Type] OR "randomized controlled trial"[Publication Type]) AND ("esophageal squamous cell carcinoma"[Title/Abstract] OR "esophageal squamous cell cancer"[Title/Abstract] OR "escc"[Title/Abstract] OR "esophageal carcinoma"[Title/Abstract] OR "esophageal cancer"[Title/Abstract] OR "esophageal neoplasm"[Title/Abstract] OR "esophageal neoplasms"[MeSH Terms]) AND ("nivolumab"[Title/Abstract] OR "opdivo"[Title/Abstract] OR "BMS-936558"[Title/Abstract] OR "MDX1106"[Title/Abstract] OR "pembrolizumab"[Title/Abstract] OR "lambrolizumab"[Title/Abstract] OR "keytruda"[Title/Abstract] OR "MK-3475"[Title/Abstract] OR "cemiplimab"[Title/Abstract] OR "libtayo"[Title/Abstract] OR "REGN2810"[Title/Abstract] OR "toripalimab"[Title/Abstract] OR "JS001"[Title/Abstract] OR "camrelizumab"[Title/Abstract] OR "SHR-1210"[Title/Abstract] OR "sintilimab"[Title/Abstract] OR "IBI308"[Title/Abstract] OR "serplulimab"[Title/Abstract] OR "HLX10"[Title/Abstract] OR "pd 1 inhibitor"[Title/Abstract] OR "pd 1 blockade"[Title/Abstract] OR "programmed cell death 1 inhibitor"[Title/Abstract] OR "anti-pd-1"[Title/Abstract] OR "anti programmed cell death 1"[Title/Abstract] OR "checkpoint inhibitor"[Title/Abstract] OR "checkpoint blockade"[Title/Abstract]) | 192 |
| Embase | #1. nivolumab:ab,ti OR opdivo:ab,ti OR 'bms 936558':ab,ti OR mdx1106:ab,ti OR pembrolizumab:ab,ti OR lambrolizumab:ab,ti OR keytruda:ab,ti OR 'mk 475':ab,ti OR cemiplimab:ab,ti OR libtayo:ab,ti OR regn2810:ab,ti OR toripalimab:ab,ti OR js001:ab,ti OR camrelizumab:ab,ti OR 'shr 1210':ab,ti OR sintilimab:ab,ti OR ibi308:ab,ti OR ('pd 1':ab,ti AND inhibitor:ab,ti) OR ('pd 1':ab,ti AND blockade:ab,ti) OR (programmed:ab,ti AND cell:ab,ti AND death:ab,ti AND 1:ab,ti AND inhibitor:ab,ti) OR 'anti pd 1':ab,ti OR ('anti programmed':ab,ti AND cell:ab,ti AND death:ab,ti AND 1:ab,ti) OR (checkpoint:ab,ti AND inhibitor:ab,ti) OR (checkpoint:ab,ti AND blockade:ab,ti) OR 'programmed death ligand 1 inhibitors':ab,ti 73,106  #2. ('esophageal squamous cell carcinoma':ab,ti OR 'esophageal squamous cell cancer':ab,ti OR escc:ab,ti OR 'esophageal carcinoma':ab,ti OR 'esophageal cancer':ab,ti) AND 'esophageal squamous cell carcinoma':de 15,500  #3. 'crossover procedure':de OR 'double-blind procedure':de OR 'randomized controlled trial':de OR 'singleblind procedure':de OR random*:de,ab,ti OR factorial*:de,ab,ti OR crossover*:de,ab,ti OR ((cross NEXT/1 over*):de,ab,ti) OR placebo*:de,ab,ti OR ((doubl* NEAR/1 blind*):de,ab,ti) OR ((singl* NEAR/1 blind*):de,ab,ti) OR assign*:de,ab,ti OR allocat*:de,ab,ti OR volunteer*:de,ab, 3,100,947  #4. #1 AND #2 AND #3 130 | 130 |
| Cochrane Central Register of Controlled Trials | #1 MeSH descriptor: [Esophageal Neoplasms] explode all trees 2191  #2 (Esophageal Neoplasms or Neoplasms, Esophagus or Esophagus Neoplasm or Esophageal Neoplasm or Neoplasm, Esophageal or Esophagus Neoplasms or Neoplasms, Esophageal or Neoplasm, Esophagus or Esophageal Cancer or Cancers, Esophageal or Cancers, Esophagus or Esophagus Cancer or Cancer, Esophagus or Esophageal Cancers or Cancer of the Esophagus or Cancer, Esophageal or Cancer of Esophagus or Esophagus Cancers or Esophageal Cancer or Esophageal Cancers or esophageal squamous cell carcinoma OR esophageal squamous cell cancer OR ESCC OR esophageal carcinoma OR esophageal cancer):ti,ab,kw (Word variations have been searched) 7104  #3 #1 or #2 7104  #4 MeSH descriptor: [Immune Checkpoint Inhibitors] explode all trees 180  #5 (nivolumab OR opdivo OR BMS-936558 OR MDX1106):ti,ab,kw (Word variations have been searched) 2713  #6 (pembrolizumab OR lambrolizumab OR keytruda OR MK-3475):ti,ab,kw (Word variations have been searched) 2704  #7 (cemiplimab OR libtayo OR REGN2810):ti,ab,kw (Word variations have been searched) 108  #8 (toripalimab OR JS001):ti,ab,kw (Word variations have been searched) 121  #9 (camrelizumab OR SHR-1210):ti,ab,kw (Word variations have been searched) 227  #10 (sintilimab OR IBI308):ti,ab,kw (Word variations have been searched) 140  #11 (Blockade, PD-1 or PD 1 Blockade or PD-1 Blockade or PD-1 Inhibitors or PD 1 Inhibitor or Programmed Cell Death Protein 1 Inhibitors or Inhibitor, PD-1 or Programmed Cell Death Protein 1 Inhibitor or PD 1 Inhibitors or PD-1 Inhibitor or Checkpoint Inhibition, Immune or Immune Checkpoint Blockade or Checkpoint Blockade, Immune or Immune Checkpoint Inhibition or Checkpoint Inhibitors, Immune or Immune Checkpoint Inhibitor or Immune Checkpoint Blockers or Checkpoint Inhibitor, Immune or Checkpoint Blockers, Immune):ti,ab,kw (Word variations have been searched) 9019  #12 #4 or #5 or #6 or #7 or #8 or #9 or #10 or #11 12372  #13 #3 and #12 355 | 355 |

## Supplementary Figure 1. Risk-of-bias assessment of the three included RCTs.


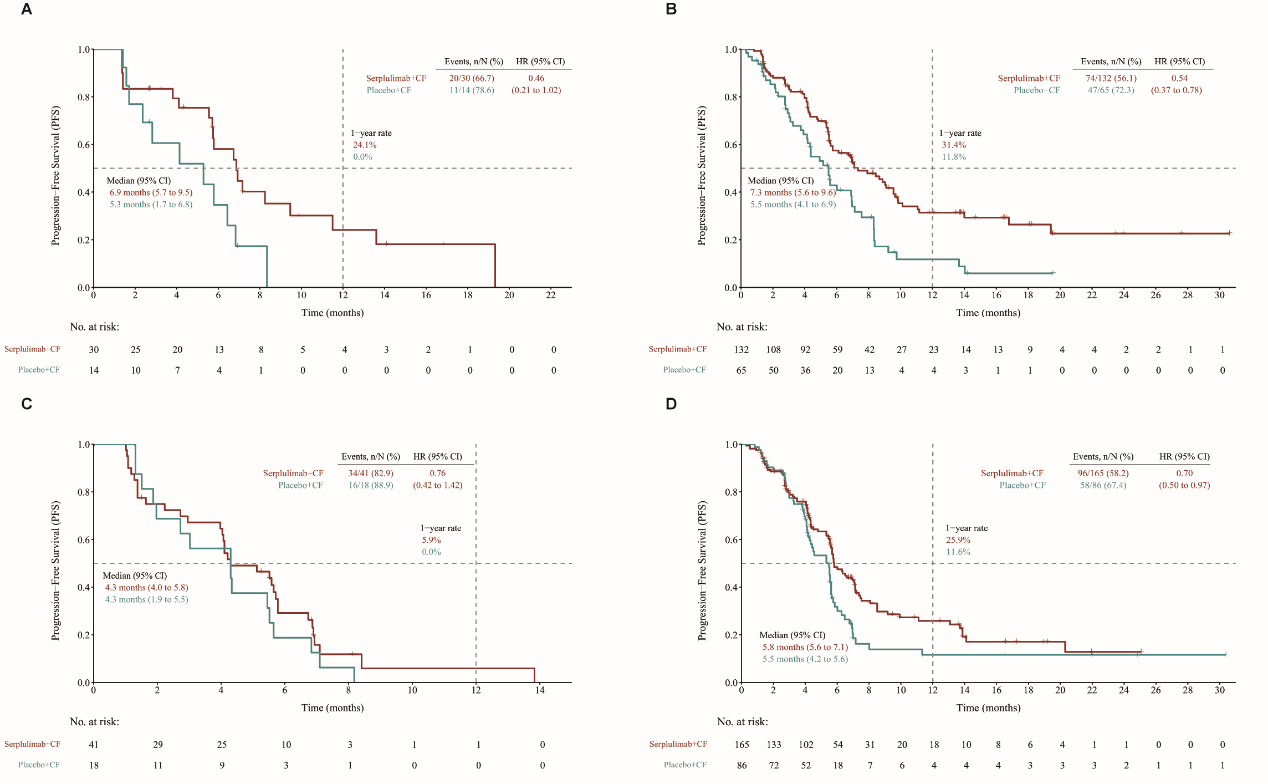


**Supplementary Figure 2. PFS in the PD-L1 expression subgroups of patients with or without liver metastases.**

Kaplan-Meier estimated PFS curves are shown to compare the serplulimab-chemotherapy group with the placebo plus chemotherapy group in patients with CPS ≥ 10 and liver metastases(A); CPS ≥ 10 and non-liver metastases (B); 1 ≤ CPS < 10 and liver metastases(C); 1 ≤ CPS < 10 and non-liver metastases(D); Patients receiving serplulimab-chemotherapy treatment are shown in red, whereas those receiving placebo-chemotherapy treatment are shown in green. CF=cisplatin and 5-fluorouracil; CI=confidence interval; HR=hazard ratio; PD-L1=programmed cell death-ligand 1; CPS=combined positive score; PFS=progression-free survival.


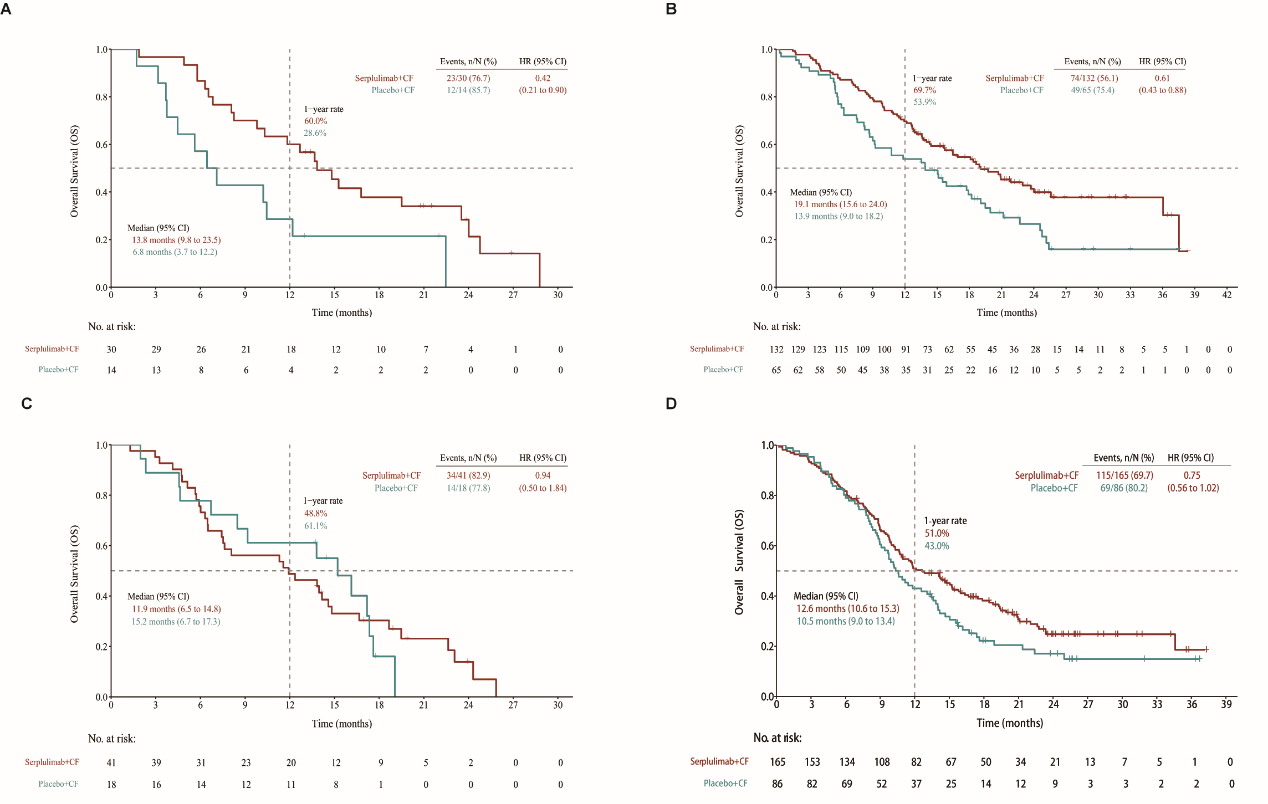


**Supplementary Figure 3. OS in the PD-L1 expression subgroups of patients with or without liver metastases.**

Kaplan-Meier estimated OS curves are shown to compare the serplulimab-chemotherapy group with the placebo plus chemotherapy group in patients with CPS ≥ 10 and liver metastases(A); CPS ≥ 10 and non-liver metastases (B); 1 ≤ CPS < 10 and liver metastases(C); 1 ≤ CPS < 10 and non-liver metastases(D); Patients receiving serplulimab-chemotherapy treatment are shown in red, whereas those receiving placebo-chemotherapy treatment are shown in green. CF=cisplatin and 5-fluorouracil; CI=confidence interval; HR=hazard ratio; PD-L1=programmed cell death-ligand 1; CPS=combined positive score; OS=overall survival.


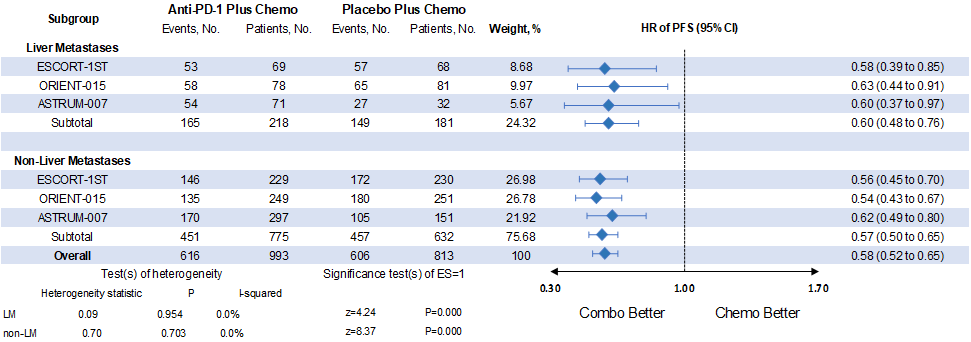


**Supplementary Figure 4. Meta-analysis of PFS with PD-1 antibody plus chemotherapy versus chemotherapy alone in the liver metastases and non-liver metastases subgroups.** The forest plot shows pooled HRs for PFS. LM = liver metastases; HR = hazard ratio; PD-1 = Programmed Death-1; Combo = Anti-PD-1 plus Chemo; Chemo = chemotherapy; ES = effect size; PFS = progression-free survival.
